# A Review of Non-Invasive Optical Systems for Continuous Blood Glucose Monitoring

**DOI:** 10.3390/s21206820

**Published:** 2021-10-14

**Authors:** Bushra Alsunaidi, Murad Althobaiti, Mahbubunnabi Tamal, Waleed Albaker, Ibraheem Al-Naib

**Affiliations:** 1 Biomedical Engineering Department, College of Engineering, Imam Abdulrahman Bin Faisal University, Dammam 31441, Saudi Arabia; 2160002911@iau.edu.sa (B.A.); mmalthobaiti@iau.edu.sa (M.A.); mtamal@iau.edu.sa (M.T.); 2 Department of Internal Medicine, College of Medicine, Imam Abdulrahman Bin Faisal University, Dammam 31451, Saudi Arabia; wialbakr@iau.edu.sa

**Keywords:** diabetes, glucose, non-invasive, optics, spectroscopy, infrared, Raman, terahertz, fluorescent, photoacoustic

## Abstract

The prevalence of diabetes is increasing globally. More than 690 million cases of diabetes are expected worldwide by 2045. Continuous blood glucose monitoring is essential to control the disease and avoid long-term complications. Diabetics suffer on a daily basis with the traditional glucose monitors currently in use, which are invasive, painful, and cost-intensive. Therefore, the demand for non-invasive, painless, economical, and reliable approaches to monitor glucose levels is increasing. Since the last decades, many glucose sensing technologies have been developed. Researchers and scientists have been working on the enhancement of these technologies to achieve better results. This paper provides an updated review of some of the pioneering non-invasive optical techniques for monitoring blood glucose levels that have been proposed in the last six years, including a summary of state-of-the-art error analysis and validation techniques.

## 1. Introduction

Diabetes mellitus is a chronic disease that affects the metabolism process. It is distinguished by abnormal levels of glucose in the blood caused by either a malfunction in the pancreas to secrete sufficient insulin due to autoimmune β-cell destruction (type 1) or inappropriate response of body cells to insulin due to a progressive loss of β-cell insulin secretion (type 2) [1,2], where insulin is the hormone secreted from the pancreas to control glucose levels in the blood. The pathophysiology of type 2 is characterized by peripheral insulin resistance, impaired regulation of hepatic glucose production, and deteriorating β-cell function, eventually leading to β-cell failure [3,4]. It is often correlated with a strong genetic predisposition or family history in first-degree relatives. There is also a gestational diabetes mellitus that occurs in the second or third trimester of pregnancy [2]. Maintaining glucose levels in the normal range is essential to avoid life-threatening complications such as renal failure, peripheral neuropathy, and cardiovascular diseases [5]. Hypoglycemia is defined as glucose levels below 70 mg/dL [6]. On the contrary, hyperglycemia is defined when the glucose level is above 120 or 180 mg/dL when fasting or after meals, respectively [6,7]. The incidence of diabetes has been increasing significantly on a global level. According to the latest statistics issued by the International Diabetes Federation, the number of adult diabetes cases reached 463 million worldwide in 2019, and it is anticipated to rise to 700 million cases by 2045 [8]. With the growing prevalence of diabetes, the economic cost has become burdensome. The total cost of US-diagnosed diabetes in 2017 was estimated by the American Diabetes Association to be $327 billion, with an average individual expense of $16,752 per year [9]. These figures demonstrate the significance of diabetes and encourage scientists and researchers to develop innovative solutions to make the lives of diabetics easier.

**Figure 1:**
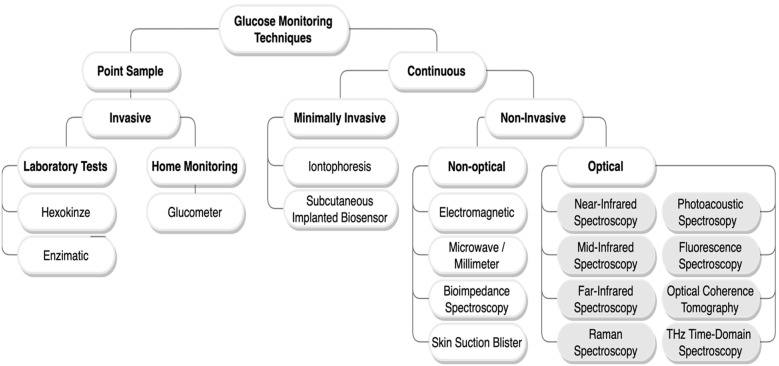
Glucose monitoring techniques classification chart. In this paper, only the non-invasive optical methods with a grey background are reviewed.

As no cure has been found for diabetes yet, regular monitoring and control of glucose concentration in the blood is the only solution to optimize the lifestyle of diabetics and prevent them from experiencing severe complications [10]. Various glucose monitoring techniques have been developed recently. These technologies are classified based on their mechanism as invasive (IN), minimally invasive (MI), and non-invasive (NI). Figure 1 maps most of the glucose-sensing technologies [6,7,8].

The invasive methods are the gold standard and the most broadly employed for blood glucose measurements [6]. Conventional devices currently in use, such as the self-monitoring blood glucose (SMBG) devices, so-called glucometer, and the continuous-glucose-monitoring (CGM) devices, follow the invasive and minimally invasive methods, respectively [11,12,13]. They are both based on electrochemical biosensors. The SMBG sensors require drawing a drop of blood to be tested through finger-pricking, while the CGM sensors are based on a needle implanted subcutaneously. Although these techniques give high accuracy measurements, they have multiple drawbacks for being painful, promoting infection, using cost-intensive blood glucose testing supplies, and deterioration of accuracy over time. Accordingly, scientists have conducted studies on non-invasive glucose sensors that are reliable, fast, painless, and cost-effective for the convenience of patients to monitor their glucose level frequently; hence, reducing diabetes complications. Various types of non-invasive techniques have been proposed during the last two decades, including non-optical and optical techniques as illustrated in Figure 1. Among the non-invasive glucose monitoring techniques, the optical methods give the best measurements [14]. Optical technologies such as near-infrared, mid-infrared, or Raman spectroscopy have great selectivity for glucose sensing given the complexity of the blood/tissue properties. Furthermore, the targeted biological tissue in optical approaches is less exposed to irritation [14]. Therefore, this paper focuses on the latest non-invasive optical sensors for blood glucose monitoring, which are depicted in Figure 1. It is worth mentioning that Table 1 contains some of the recent review papers addressing the non-invasive glucose monitoring techniques. Specific criteria were followed to review the techniques. Only research papers that were published between 2015 to 2021 and included optical approaches were reviewed.

## 2. Non-Invasive Optical Techniques

In this section, seven optical techniques are briefly explained and recent developments of each of them are reviewed accordingly. For most of them, a summary of their features, advantages, and limitations is included.

### 2.1. Near-Infrared (NIR) Spectroscopy

Near-infrared (NIR) spectroscopy is a spectrophotometric technique used to obtain quantitative data about a sample of biological tissue and its constituents. It employs light in the 750–2500 nm wavelength range of the electromagnetic spectrum in which the light can penetrate the skin beyond 0.5 mm with low-energy radiation. The existence of C-H, O-H, and C=O bonds in glucose molecules increases the NIR light absorption in the blood, which peaks at specific wavelengths. Mathematically, the Beer–Lambert law is used for the formulation, which allows the calculation of absorbance of a sample from the concentration and the thickness of the sample [18]. For more detail, the reader is referred to Refs. [19,20,21]. In contrast to other optical techniques, NIR spectroscopy has the advantage of measuring glucose without prior manipulation needed in an affordable and compact manner. Due to the considerably high penetration level, NIR measurements can be accomplished using the transmission and reflection modes [15]. Blood glucose levels are estimated by detecting the variations in the light intensity caused by the absorption or scattering of glucose molecules to NIR light, which depends on the concentration of the glucose in the tissue [22].

**Figure 2:**
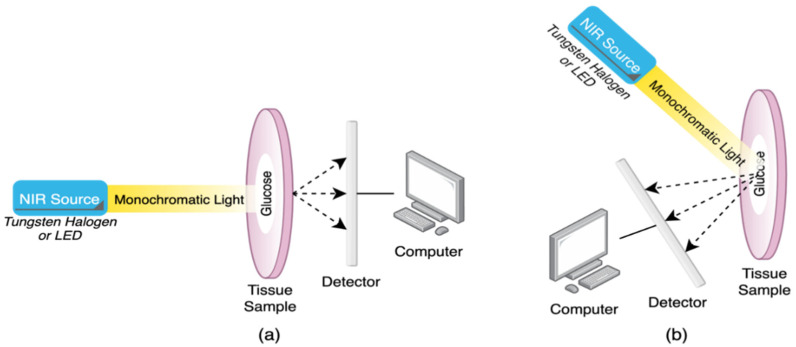
Schematic representation of (**a**) transmission and (**b**) reflection configurations of NIR spectroscopy. In the transmission configuration, the detector is located on the other side of the sample and only the transmitted photons are measured. In the reflection configuration, the detector is located on the same side of the sample and only the scattered photons are measured.

The NIR spectroscopy setup consists mainly of a NIR source with a specific wavelength, a tissue sample subjected to NIR radiation, and a photodiode to measure the attenuated light waves that are either transmitted or reflected from the tissue sample to the detector. Figure 2 includes simplified diagrams of the transmission and reflection modes of NIR spectroscopy.

**Figure 3:**
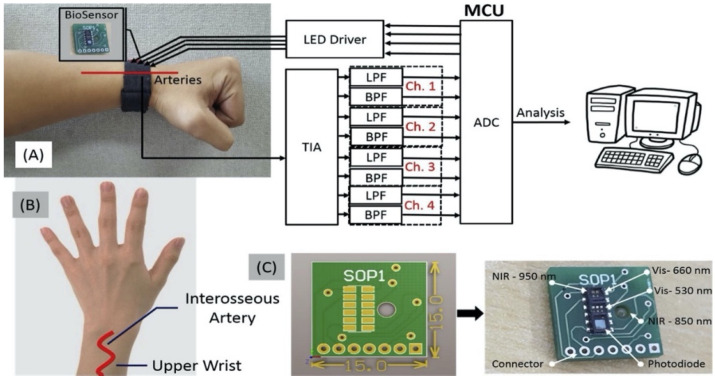
A wearable glucose sensor system: (**A**) signal processing block diagram, (**B**) measurement site, and (**C**) designed printed circuit board with four photodiodes biosensor. Reprinted with permission from [23].

The choice of the system setup depends on the type of specimen. If the sample is thick and dense, the reflection configuration is preferable, while the transmission configuration gives better results with aqueous and thin samples [5]. Moreover, the transmission configuration is restricted to certain areas of the human body such as the fingertip and earlobe. In contrast, the reflection configuration has the advantage to allow for measurements from a wider selection of human body parts such as forearm, forehead, cheek, fingertip, and earlobe. Here, we highlight two recent studies, one utilizing the reflection configuration while the other exploited the transmission mode. A recent study by Rachim et al. designed a wearable optical glucose biosensor that uses multiple light sources in the visible (Vis) to NIR region. The device reads the glucose measurements from the wrist of the hand through a diffused-reflection setup. Figure 3 shows the prototype of the proposed system that uses four channels with wavelengths of 530 nm, 660 nm, 850 nm, and 950 nm [23]. The system features a compact footprint of 15 × 15 mm^2^ that can be worn around the wrist with a sensitivity of 6.16 mg/dL and a correlation coefficient (R^2^) of 0.86. The experiment was done in-vivo, and the number of the subjects was limited to 12.

**Figure 4:**
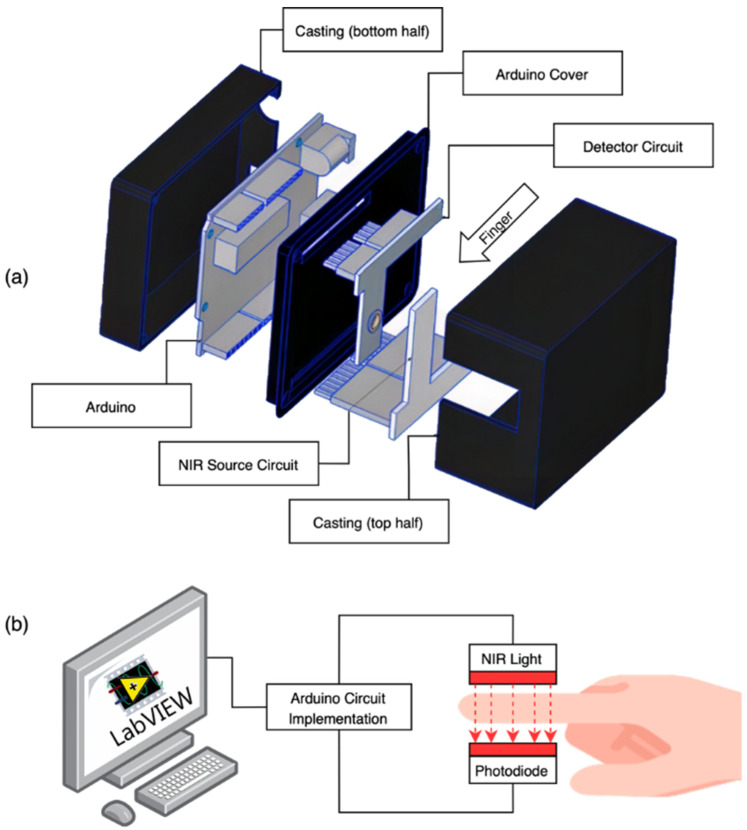
(**a**) A prototype for the NIR transmission spectroscopy for the noninvasive glucose monitoring system and (**b**) the finger placement illustration. In this prototype, only transmitted photons via the finger are measured. Reproduced with permission from [24].

Another recent study introduced a prototype for NIR transmission spectroscopy for the noninvasive glucose monitoring system [24]. Figure 4 illustrates the setup for this design. Here, a light beam passes through the human fingertip. The design uses a photodiode light source at the operating wavelength of 940 nm and a Silicon PIN Photodiode is used for the detection of the transmitted light. This work has experimentally verified the relationship between the sensor output voltage and the glucose concentration. The device was tested on five volunteers to determine the glucose level in the blood. The results were benchmarked with invasive devices for each volunteer and there was a good agreement between both methods.

**Figure 5:**
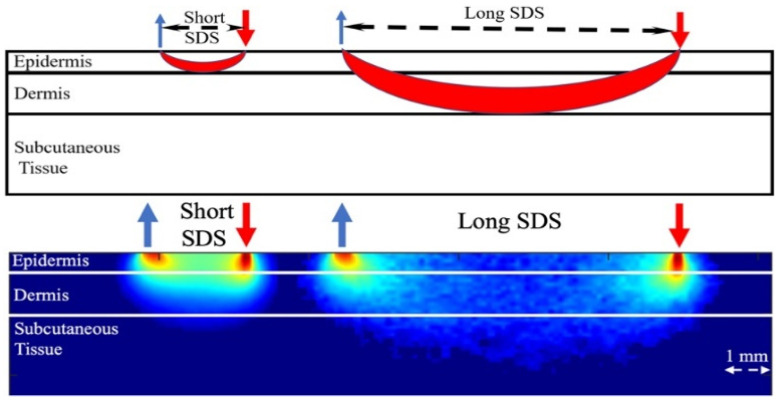
Dual-channel near-infrared sensor where the SDS for the short channel (**left side**) is 2 mm at 1450 nm and the SDS for the long channel (**right-side**) is 6 mm at 1750 nm. Reprinted with permission from [25].

More recently, a dual-channel approach for NIR sensors is introduced where four optodes for short and long channels are utilized [25]. Figure 5 shows the proposed dual-channel near-infrared sensor. The signal from the long channel includes significant information about the glucose level while the short channel is utilized to measure the interference “noise” signal originating from the epidermis layer. The former signal can be utilized to calculate the glucose contents in the dermis layer of the skin and the latter can then be removed from the long channel signal. The two sources operate at two different wavelengths. The source-detector separation is calculated based on an optimized module.

**Figure 6:**
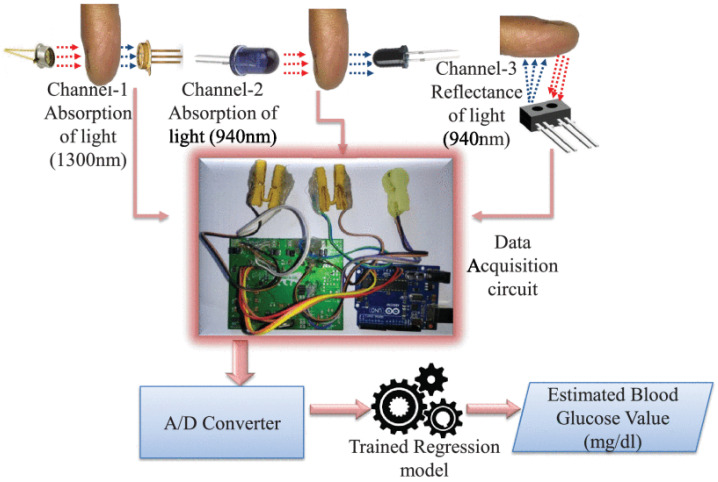
Illustration of the proposed iGLU 2.0 device using dual NIR spectroscopy that involves absorption and reflection spectroscopy of 940 nm, and absorption spectroscopy of 1300 nm. Reprinted with permission from [26].

Furthermore, the authors of Ref. [26] proposed a NIR serum glucose level monitoring system using absorption and reflection-based dual NIR spectroscopy and calibrated machine learning models. It is a new non-invasive wearable device that measures serum glucose. Figure 6 illustrates the proposed device, which consists of three channels: The first channel uses a transmission configuration to measure the absorption of LED 1300 nm. The second and the third channels involve absorption and reflection spectroscopy of LED 940 nm. Deep neural network and polynomial regression models were utilized for serum glucose prediction. The device has been validated by collecting samples of both capillary glucose and serum glucose from prediabetic, diabetic, and healthy subjects. The device showed high accuracy serum glucose prediction in comparison with other NIR measurement techniques. The ability of this device to be compatible with the Internet of Medical Things framework and to accurately measure serum glucose are great advantages.

### 2.2. Mid-Infrared (MIR) Spectroscopy

**Figure 7:**
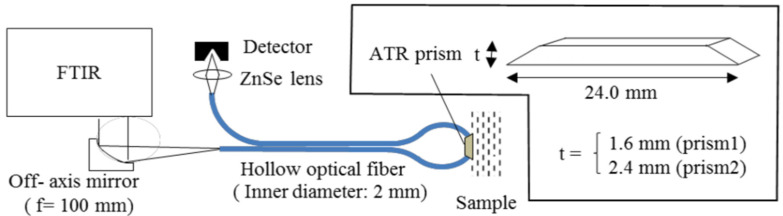
Schematic setup of the proposed MIR system, where the light is propagated via a hollow fiber to an ATR prism that is put inside the mouth. Accordingly, the absorbance of the oral mucosa is measured using two ATR prisms of different thicknesses. Reprinted with permission from [27] © The Optical Society.

As with NIR spectroscopy, MIR spectroscopy is used to acquire numerical information about a sample based on the absorption spectra. Nevertheless, MIR utilizes a longer wavelength of light in the region of 2500–10,000 nm that covers the fingerprint region of glucose. Consequently, less scattering and higher absorption occur in the tissue, resulting in distinct and sharp peaks in the absorption spectra of glucose and other chromophores, unlike the NIR band response that gives weak and broad peaks [16,22]. However, MIR can only penetrate the skin to a minimal range of about 100 μm due to the strong absorption of water and other biological compounds to MIR. This limits the system configuration to the reflection setup since it is unachievable to obtain transmission measurements [5]. The MIR spectroscopy has the same reflection mode as the NIR spectroscopy shown in Figure 2b. Kasahara et al. proposed a method in 2018 to estimate glucose concentration by measuring the absorbance of the oral mucosa to a MIR light source using hollow fibers and attenuated total reflection (ATR) prism. The absorbance is measured from the detected beam coming from the ATR prism, which is placed in between the upper and lower lips, through the hollow fiber. A simplified representation of the system is shown in Figure 7 [27]. The experiment was carried out in-vivo on five subjects and the correlation coefficient was 0.36.

### 2.3. Raman Spectroscopy

**Figure 8:**
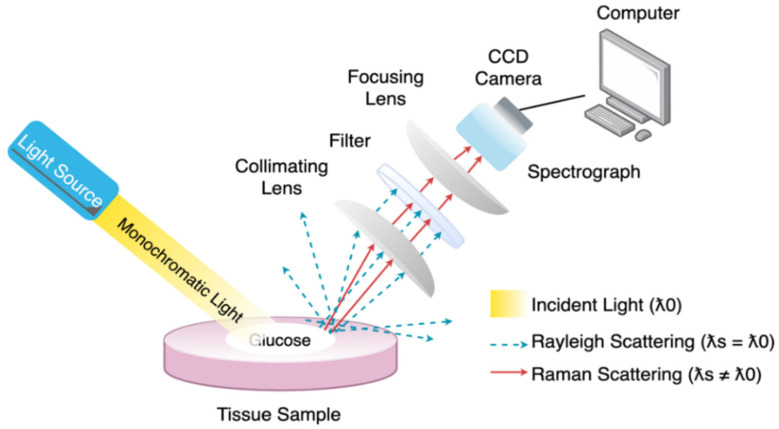
A simplified diagram of Raman spectroscopy where a collimating lens capture part of the scattered radiation and directing it to a filter, so only the Raman scattered light at a wavelength that is different from the incident light to be sensed by the detector. The computer processes the signal and provides the corresponding Raman shift. Redrawn from reference [5].

Raman technique uses a monochromatic light source ranging from visible to MIR to detect the glucose concentration based on the Raman effect. When monochromatic light strikes the tissue sample, it produces scattered rays that travel in all directions. Most of these rays come out with the same wavelength as incident light and are called elastic or Rayleigh scattering. The rest of the beams are inelastic scattering, also known as Raman scattering, with different wavelengths from the incident light due to interaction with the tissue sample causing rotation and vibration of the molecules. The resulting difference in the wavelength is the Raman shift. This shift provides information about body fluids’ rotational and vibrational states to monochromatic light. The vibration between the molecules is dependent on the molecular concentration of the human fluids. Accordingly, the estimation of glucose concentration is feasible [28]. What makes this spectroscopy superior are the distinctive narrow peaks shown in the band response, which facilitate the separation of the signals [29]. Figure 8 illustrates the system configuration of Raman Spectroscopy.

**Figure 9:**
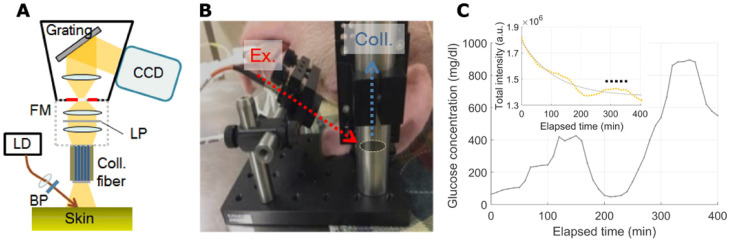
The suggested system: (**A**) schematic diagram, (**B**) experiment setup, and (**C**) obtained glucose profile. Exponential time decay of fluorescence from the skin in the inset (actual data in the yellow dotted line and its exponential approximation in the gray line). Reprinted with permission from [30].

A recent in vivo study by Kang et al. developed an off-axis portable instrument for non-invasive glucose monitoring using Raman spectroscopy [30]. A laser diode (LD) of 830 nm was targeted at the ear tissue of the animal. Figure 9 shows the simplified diagram of the proposed system, the setup of the experiment, and the glucose profile obtained during the experiment [30]. The exact value of the correlation coefficient was not determined due to the limited measured data points. Nevertheless, the minimum detectable concentration was estimated to be between 29 and 78 mg/dL.

### 2.4. Far Infrared (FIR) Spectroscopy and Terahertz Time-Domain Spectroscopy (THz-TDS)

FIR spectroscopy, also known as terahertz spectroscopy, relies on the interaction between the electromagnetic field of light and the electric/magnetic dipoles of matter. These interactions produce vibrational and rotational transitions of weak bonds and bonds of large atoms with wavelengths ranging from 10 μm to 1000 μm, which are equivalent to 0.3 THz to 30 THz [31,32,33,34]. Changes in the transmission of FIR radiation through the sample yield data about the sample and allow us to understand its characteristics. However, because of the strong absorption of water and due to the low level of penetration, the estimation of glucose measurements using standard FIR spectroscopy is difficult. Instead, it is feasible to use terahertz time-domain spectroscopy (THz-TDS) for this purpose [35,36,37,38]. In this approach, the phase transition along with the amplitude information is recorded. When the light travels across the specimen, some photons will transmit to the detector directly. However, others will take a longer path or will not even reach the photodetector due to the optical properties of biological tissues, including internal reflection and scattering.

**Figure 10:**
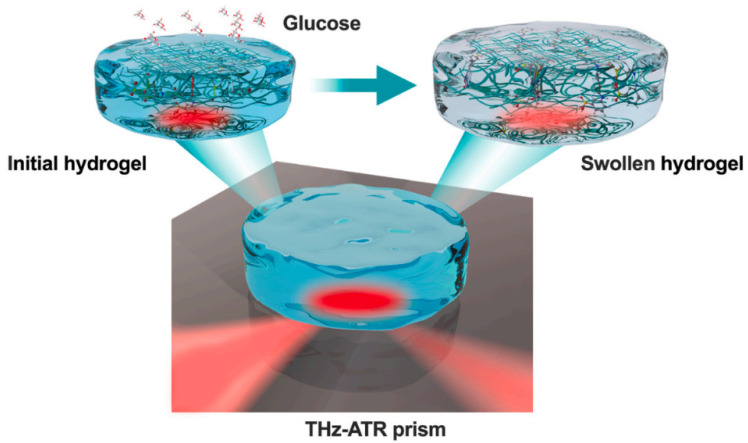
THz-ATR setup schematic with a smart hydrogel where the incident THz wave from the bottom left-hand side is propelled to the upper interface of the THz-ATR prism in the middle and then generates evanescent waves that penetrate the sample attached to the surface of the prism. The output reflected THz signal shown on the bottom right-hand side contains information about the permittivity of the sample. Hence, the complex permittivity of the samples can be obtained. Reprinted with permission from [45].

The glucose concentration and other optical properties can be extracted from evaluating the disruption of photons’ time of flight, the shape of pulses, and the glucose absorption rates [39,40,41,42,43,44,45]. For instance, a sugar concentration of a few hundred micromoles was sensed using a nanoslit antenna [43]. Moreover, a linear relationship in 70 diabetic patients between the blood glucose level and THz absorbance has been demonstrated [42]. An error rate lower than 15% was found in 20 blood samples. More recently, THz attenuated total reflection (THz-ATR) has been utilized to probe the glucose-induced hydration level of smart hydrogels for selective detection of aqueous glucose [45]. A schematic of the sensing setup is shown in Figure 10. It consists of a THz-ATR prism and a smart hydrogel that is responsive to glucose. In this setup, the THz signal is directed to the upper interface of the prism and then generates evanescent waves that penetrate the sample attached to the surface that will propagate tens of micrometers inside the sample. The THz-ATR device could quantitatively and selectively detect aqueous glucose with quite good sensitivity that would not be achievable using conventional THz spectroscopy [45]. The experiment was done in-vitro and the sensor showed a linear response for a glucose concentration level up to 200 mg/dL with a correlation coefficient of 0.961.

### 2.5. Fluorescent Spectroscopy

**Figure 11:**
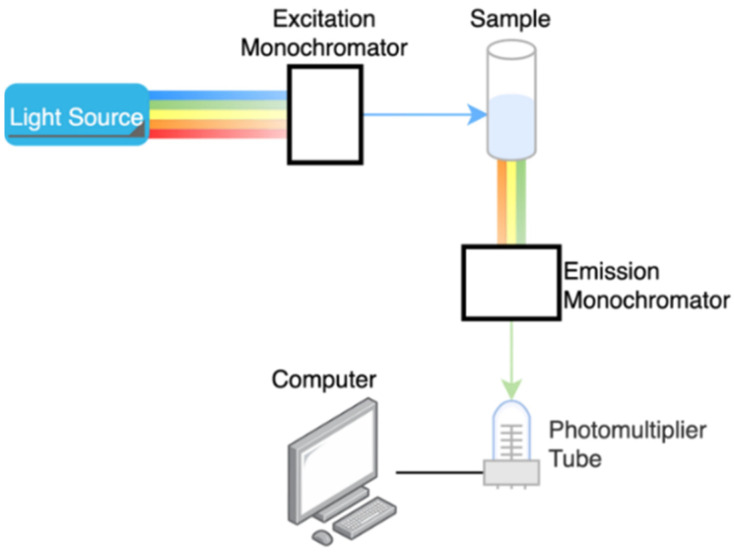
Schematic diagram of fluorescent spectroscopy.

The human body naturally contains various fluorescent molecules known as intrinsic fluorophores. These fluorophores can emit light at a specific wavelength when it is excited by incident radiation at a higher energy state. This difference between the wavelengths is called the Stokes shift. The emitted light has distinct properties equivalent to the concentration of the analyte under study. Studies show that glucose can emit fluorescent light if exposed to visible or ultraviolet light. However, it has issues in signal production and interference. Therefore, to measure glucose concentration, it is proposed to use a fluorescent labeling method [46], which binds glucose with an exogenous fluorophore called a receptor. The experiment in this work was done in-vitro with 60 samples and the achieved correlation coefficient was found to be 0.964. These are engineered fluorophores designed to fluoresce only in the presence of glucose, which helps in decreasing electron sharing and increasing fluorescence. This technique allows for extremely sensitive measurements. Nevertheless, the fluorescence intensity depends on various parameters, including the glucose concentration, skin pigmentation, redness of the skin, and thickness of the epidermis layer; hence, the radiation of ultraviolet light on the tissue causing strong scattering [13,47,48]. The fluorescent spectroscopy system setup is shown in Figure 11.

### 2.6. Photoacoustic Spectroscopy (PAS)

**Figure 12:**
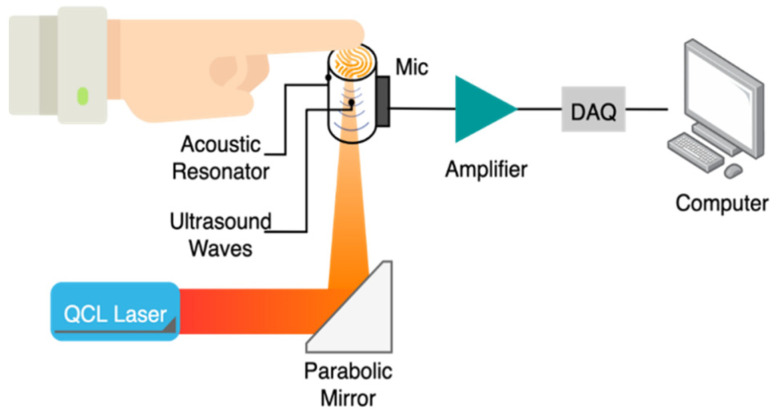
System diagram of photoacoustic spectroscopy for non-invasive glucose measurements. Redrawn from reference [5].

PAS is a technique that relies on the acoustic waves generated following the emission of short laser pulses through the sample to measure the concentration of its chromophores. The radiation of a specific wavelength hits the targeted tissue sample for a short time in the range of nanoseconds. Consequently, the sample constituents absorb the optical energy and release it as heat causing expansion in the volume of the subjected medium. Due to volumetric changes, ultrasound waves were generated. These ultrasound waves can be measured at the surface of the sample using a piezoelectric transducer. The changes in blood glucose levels can be determined by monitoring the variations in the peak-to-peak of the detected signal [22,49]. The system of PAS consists mainly of a laser diode, resonator, transducer, projection system, and microcontroller. Figure 12 presents the system configuration of PAS sensing. The ultrasonic waves generated by the emission of light through the sample propagates through the acoustic resonator. Then, it reaches the detector, which is generally made of a piezoelectric transducer like a microphone. The electrical signal at the sensor output is later amplified, digitized, and sent to a computer for analysis [5]. In a recent work [14], a method to solve the challenges of photoacoustic spectroscopy for non-invasive glucose monitoring was presented by obtaining the microscopic spatial information of skin during the spectroscopy measurement. The field of view of photoacoustic images of the skin was 1.3 mm by 1.3 mm and the skin is exposed to the detector in a 2.5 mm diameter. A total of 70% of the measured points fell into zone A of Clarke’s grid and 30% into zone B for a total of 76 measurements that were acquired from the pooled data from the five independent experiments with a healthy and diabetic subject.

**Figure 13:**
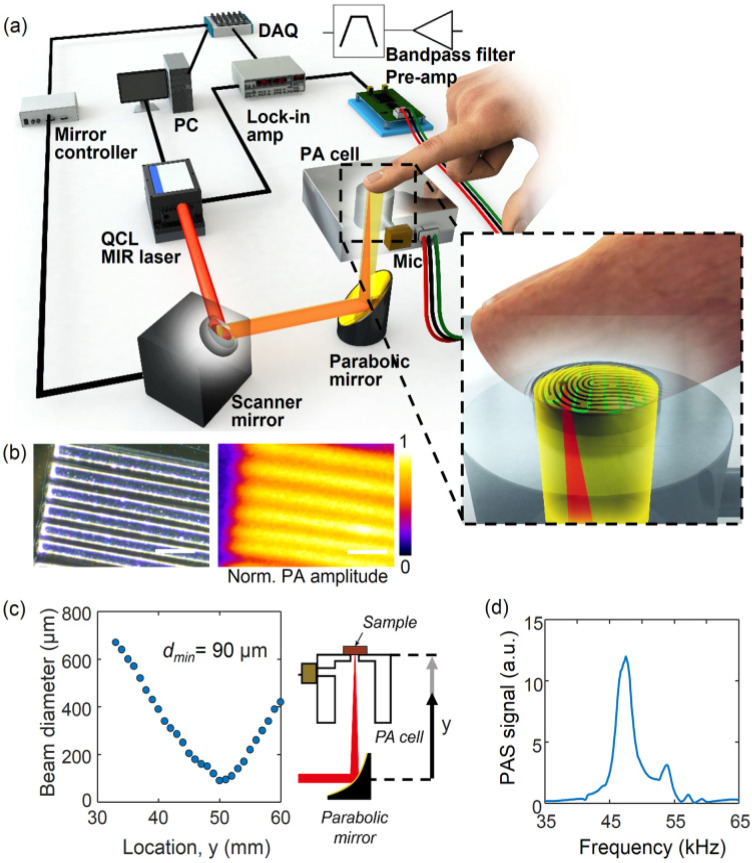
Photoacoustic spectroscopy system: (**a**) schematic diagram, (**b**) optical micrograph and photoacoustic image of SU-8 structures for resolution evaluation, (**c**) reflected beam diameter from the parabolic mirror, and (**d**) PAS peak signal that is located at 47.5 kHz with a reference carbon black tape sample. Reprinted with permission from [14].

Sim et al. presented an in vivo trial to overcome the hurdles that are facing photoacoustic spectroscopy-based non-invasive glucose sensors [14]. The idea was to select a skin region where the readings of mid-infrared spectra are unaffected by skin conditions such as sweating. It was achieved by identifying the microscopic spatial information of the skin during the experiment. This increased the reliability of blood glucose level estimation. Figure 13 represents the system diagram, a micrograph image that helps in resolution, the diameter of the reflected beam, and the peak signal [14].

### 2.7. Optical Coherence Tomography (OCT)

Optical coherence tomography (OCT) is an optical technique that can non-invasively measure real-time, 3D tissues images with very fine spatial resolution, approximately 1–15-μm based on the specific used OCT technique. OCT measures both the intensity and time-of-flight information of light photons that are scattered back from different locations within a tissue [50,51]. This information is then used to reconstruct 3D depth-resolved tissue information. The OCT technique has been widely utilized for various clinical applications [52,53,54,55]. In the field of noninvasive glucose monitoring, OCT has been extensively investigated. The work of [56] presented the linear relationship between blood glucose concertation and the slope of the OCT signals. Moreover, early clinical studies have also presented the correlation between blood glucose concertation and the slope of the OCT signals [57,58]. The authors of Ref. [59] presented two approaches for blood glucose monitoring with OCT. The spatial approach is based on spatial analysis of the total attenuation coefficient while the temporal approach uses speckle decorrelation analysis.

**Figure 14:**
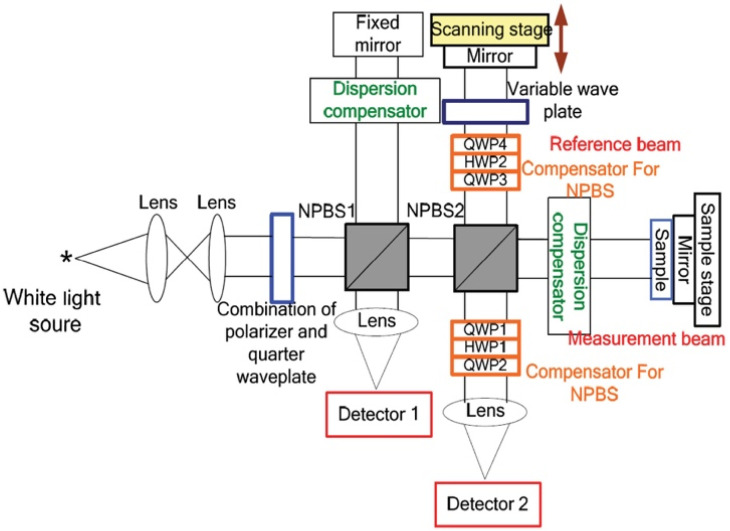
Schematic illustration of the Mueller OCT system consisted of a halogen lamp, two photo-detectors, a scanning stage, a scanning stage driver, a dispersion compensator, an oscilloscope, and two nonideal beam splitters. Compensation for the polarization distortion was performed using a composite polarizer component comprising a quarter-wave plate, half-wave plate, and second quarter-wave plate. Reprinted with permission from [62].

The work of [60] investigated the viscosity of turbid colloidal glucose solutions by the spectral domain OCT. In this study, the authors measured the viscosity of aqueous glucose solutions over a range of glucose concentrations. An increase in blood glucose concentration results in an increase in blood plasma viscosity and thus an increase in the whole blood viscosity. This study has demonstrated the potential of OCT to measure the blood viscosity, but it should be noted that glucose concentration is not the main parameter that altered blood viscosity, although it does contribute. Thus, this approach needs to be clinically demonstrated to be an adopted noninvasive glucose monitoring technique. Recently, the promising results Refs. [61,62] had led to propose a Mueller OCT system shown in Figure 14 for noninvasively measuring the glucose concentration on the human fingertip. This technique is based on detecting the optical rotation angle and depolarization index of fingertip tissue. The results show that as glucose concentration increases, the optical rotation angle linearly increases, and the depolarization index of tissue decreases. The optical rotation angle represents the circular birefringence property of the glucose sample, whereas the depolarization index represents the scattering effects caused by particles within the tissue. Both parameters are essential to have trustworthy estimates of the glucose concentration. This study had performed both phantom experimental and clinical studies and the results obtained for both are in a good qualitative agreement. The experiment was carried out in-vitro with six samples and in vivo on four subjects and the achieved correlation coefficient was found to be 0.91 for the latter. Nevertheless, further studies of people of different ages, races, and skin colors are needed to have trustable results for glucose concentration extraction.

## 3. Comparison

Despite the substantial development of glucose monitoring technologies, there is still a tremendous need for high accuracy, easy-to-use, and affordable techniques that can replace standard devices. The proposed methods face many challenges, such as system stability, sensitivity, specificity, and calibration. Among the non-invasive optical techniques discussed, PAS, fluorescence, and specifically NIR spectroscopy were the potential candidates for achieving the goal of obtaining optimal glucose sensing. Table 2 summarizes various non-invasive optical techniques for blood glucose concertation estimation.

## 4. Regulations and Error Analysis

For a device to be placed on the market, it must meet the required regulations and standards. The International Organization for Standardization (ISO) released a standard in 2013, ISO 15197, for in-vitro glucose monitoring instruments and self-monitoring glucometers to ensure the safety of the device and its suitability for human use. The system’s accuracy in measuring blood glucose levels is the main point in the regulation of glucose monitors. Several tools are used nowadays to evaluate the accuracy of a non-invasive glucose monitor [64]. The mean average relative difference, Clarke error grid (CEG), and consensus error grid, also known as Parkes error grid (PEG), all are used for accuracy assessment [65]. The mean average relative difference (MARD) is widely used for accuracy assessment in most developed glucose meters due to its simplicity [26,66]. It is expressed as a percentage and calculated by taking the average of the percentage errors between the measured value to a reference value. MARD shows how close is the measured value from the actual value. The smaller the percentage, the more accurate the device is [13,67].

**Figure 15:**
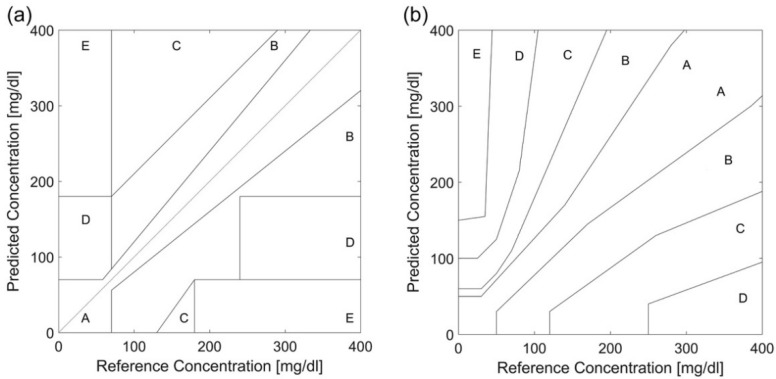
Error grids plots: (**a**) CEG and (**b**) PEG for glucose monitoring. Reprinted with permission from [13].

The error grids, CEG and PEG, are qualitative tools to measure the accuracy of non-invasive glucose monitors. The predicted glucose measurements from the device are plotted corresponding to the invasive measurements’ method. These error grids are divided into five risk areas based on the clinical significance of diabetes conditions, as shown in Figure 15 [13]. The difference is that PEG analysis differentiates between type 1 diabetes and type 2 diabetes. Table 3 describes the risk assessment for each zone in CEG analysis and PEG analysis [5,13,67].

Particular guidelines for accuracy are required to approve a self-monitoring blood glucose device. Each agency has its regulations. Table 4 lists the required criteria of both the U.S. Food and Drug Administration and the European Medicines Agency.

The performance of the non-invasive continuous blood glucose monitoring devices is usually validated against the invasive methods that are considered the gold standard. An increase in blood glucose level is typically preceded by either inflammation (acute or chronic) in the islets of Langerhans located in the pancreas, or due to insulin resistance [69]. To the knowledge of the authors, in-vivo medical imaging-based validation of those devices has not been investigated.

A recent study was carried out to correlate the semiquantitative parameter of standardized uptake value (SUV) of ^18^FDG-PET as a biomarker for monitoring low-grade beta-cell inflammation in the pancreas causing insulin deficiency [70]. Another study investigated the relationship between plasma glucose level and glucose uptake by different organs (e.g., muscle, liver, lungs, etc.) to determine insulin resistance [71]. Such an in-vivo medical imaging study could provide a detailed insight into the variations of sensitivity of non-invasive blood glucose monitoring devices and also could stratify their performances to determine the cause of elevated blood glucose levels (i.e., insulin deficiency or insulin resistance). However, the correlation between beta-cell inflammation and changes in plasma glucose level monitored by the non-invasive glucose monitoring device has not been investigated and remains an open area of research.

## 5. Commercial Devices

Hitherto, many non-invasive glucose monitors have been developed. Some of these devices have shown promising outcomes and have been successfully introduced to the market. However, due to some issues, including accuracy errors, they were dropped. Nevertheless, manufacturing companies are working on improving their devices. Table 5 lists commercial non-invasive blood glucose monitoring devices that use the optical techniques addressed in this paper. The choice to evaluate the accuracy of the device depends on the manufacturer. Some use Clarke error grid analysis (EGA) while others use consensus error grid (CEG) analysis or the mean absolute relative difference (MARD).

## 6. Conclusions

Over the past decades, there has been great interest in developing innovative methods of measuring blood glucose levels without the necessity for blood samples. Several non-invasive optical glucose measurement techniques have been introduced. These technologies still need improvement in order to meet the regulations to be released in the market. This paper provided a technical view of some of the prevalent non-invasive optical approaches in glucose sensing currently under study. It also discussed the system configuration of each technique. A summarized comparison was made on the advantages, disadvantages, and other specifications of the non-invasive optical methods discussed. Although these methods show great potential, some challenges are facing them including sensitivity, stability, specificity, biological factors, and calibration issues. Therefore, an enhancement of these non-invasive optical methods is required to surmount their limitations and hopefully replace the conventional methods currently in use.

## Figures and Tables

**Table 1:** Summary of some review papers most pertinent to non-invasive glucose sensing technologies.

Author	Main Topics	Year	Ref.
Shokrekhodaei et al.	Review of non-invasive glucose measurements methods based on glucose intrinsic properties and blood/tissue characteristics to light, and respiration acetone analysis	2020	[15]
Zhang et al.	Review of non-invasive glucose measurement methods based on electromagnetic waves	2019	[16]
Villena Gonzales et al.	Review of invasive to minimally invasive and non-invasive glucose measurement methods, available commercial devices, accuracy assessment standards, the latest glucose sensors under development, and glucose estimation informatics.	2019	[5]
Jernelv et al.	Review of non-invasive continuous glucose measurement methods based on optical technologies, challenges facing these techniques, and current research status and prospects.	2019	[13]
Delbeck et al.	Overview of glucose measurement methods with a focus on Raman, mid-infrared, and near-infrared spectroscopies studies. Review of studies on photoplethysmography technology accompanies time resolution, wavelength dependence, and spatial resolution.	2019	[17]
Bruen et al.	Review of non-invasive continuous glucose measurement methods based on the estimation of glucose concentration in other physiological solutions rather than blood.	2017	[10]

**Table 2:** Comparison of various non-invasive optical glucose sensing techniques [14,23,27,30,45,46,62,63].

Technology	Wavelength	Selectivity	Measurement Site	Merits	Drawbacks
NIR spectroscopy	750–2500 nm	Good	Ear lobe, finger, forearm, cheek, lip mucosa, oral mucosa, and tongue	- Low-cost - Easy to implement	- Glucose heterogeneous distributions affect accuracy. - Interferences by other chemical compounds
MIR spectroscopy	2500–10,000 nm	Good, superior to NIR	Finger, skin, and oral mucosa	- Quite accurate - Lightweight - Scattering is low	- Poor skin penetration depth - Expensive - High water abortion
FIR spectroscopy	10–1000 μm	Good	ISF	- Scattering is lower than NIR and MIR - Individual daily calibration is not required	- Difficulty in identifying other molecules than water due to strong water absorption
Raman spectroscopy	Visible light	Excellent	Eye, human skin	- Low sensitivity to water and temperature changes - Great specificity - Low-cost	- Lack of stability in the laser wavelength and intensity - Spectrum acquisition takes time
THz-TDS	30 µm to 3 mm	Good	ISF	- Not affected by background noise.	- Long measuring time - Low spatial and depth resolution
Fluorescence	Ultraviolet light, visible light	Excellent	Tears, human skin	- High sensitivity and specificity to glucose concentration - Not affected by light scattering	- Sensitive to changes in pH and oxygen levels - Susceptible to toxicity problems
PA spectroscopy	Ultraviolet light, NIR, and MIR	Good	Finger, forearm, and aqueous humor	- Unsusceptible to water distortion - Not affected by scattered particles	- Low signal-to-noise ratio - Affected by temperature changes, motion, pulsation, and acoustic noise

**Table 3:** Error Grids Analyses [5,13,28].

Risk Zones	CEG Analysis	PEG Analysis
Zone A	Clinically valid treatment	No effect on clinical treatment
Zone B	Clinically uncritical treatment	Mild effect on clinical treatment
Zone C	Unnecessarily treatment	Possible to affect clinical treatment
Zone D	Dangerous fails to diagnose and treat	Serious medical risks
Zone E	Extremely dangerous lead to wrong treatment	Dangerous consequences

**Table 4:** Approval Criteria for Self-monitoring Blood Glucose Devices.

Agency	Glucose Level	Min. Accuracy	Ref.
U.S. Food and Drug Administration (FDA)	Full range	95 ± 15% 99 ± 20%	[68]
European Medicines Agency (EMA)	≥100 mg/dL	95 ± 15%	[67]

**Table 5:** Summary of the commercial devices that are based on the reviewed non-invasive glucose sensing techniques.

Device	Company	Technology	Target Site	Accuracy	Approval	Ref.
TensorTip Combo Glucometer (CoG)	Cnoga Medical	VIS-NIR Spectroscopy Consists of four LEDs (625, 740, 850, 940 nm) and a color image sensor.	Fingertip	PEG: Zone A (96.6%) Zone B (3.4%) MARD: (14.4%)	CE approved FDA pending	[72,73,74]
Wizmi	Wear2b Ltd.	NIR Spectroscopy	Wrist	CEG: Zone A (93%) Zone B (7%) MARD: (7.23%)	Proof of concept	[75]
HELO Extense	World Global Network	NIR Spectroscopy	Fingertip	N/A	CE approved Discontinued	[5,76]
LTT	Light Touch Technology Ltd.	MIR Spectroscopy Uses a solid-state laser in the MIR region (6000–9000 nm) and optical parametric oscillation technology	Fingertip	N/A	Under development	[15]
NBM-200G	OrSense	Occlusion NIR Spectroscopy	Finger	CEG: Zone A (69.7%) Zone B (25.7%) MARD: (17.2%)	Discontinued	[77,78]
Eversense CGM	Senseonics	Fluorescence technology	Upper arm	MARD: (8.5%)	CE approved FDA approved	[79,80]
C8 MediSensors		Raman Spectroscopy	Abdomen	N/A	CE approved (Never released)	[78]

## Data Availability

Not applicable.
